# Functionalized Particles Designed for Targeted Delivery

**DOI:** 10.3390/polym13122022

**Published:** 2021-06-21

**Authors:** Teresa Basinska, Mariusz Gadzinowski, Damian Mickiewicz, Stanislaw Slomkowski

**Affiliations:** Centre of Molecular and Macromolecular Studies, Polish Academy of Sciences, Sienkiewicza 112, 90-363 Lodz, Poland; mariuszg@cbmm.lodz.pl (M.G.); dmi@cbmm.lodz.pl (D.M.)

**Keywords:** functional polymer, microparticle, nanoparticle, polymerosome, nucleic acid, protein, targeted drug delivery

## Abstract

Pure bioactive compounds alone can only be exceptionally administered in medical treatment. Usually, drugs are produced as various forms of active compounds and auxiliary substances, combinations assuring the desired healing functions. One of the important drug forms is represented by a combination of active substances and particle-shaped polymer in the nano- or micrometer size range. The review describes recent progress in this field balanced with basic information. After a brief introduction, the paper presents a concise overview of polymers used as components of nano- and microparticle drug carriers. Thereafter, progress in direct synthesis of polymer particles with functional groups is discussed. A section is devoted to formation of particles by self-assembly of homo- and copolymer-bearing functional groups. Special attention is focused on modification of the primary functional groups introduced during particle preparation, including introduction of ligands promoting anchorage of particles onto the chosen living cell types by interactions with specific receptors present in cell membranes. Particular attention is focused on progress in methods suitable for preparation of particles loaded with bioactive substances. The review ends with a brief discussion of the still not answered questions and unsolved problems.

## 1. Introduction

In the most desirable situation, bioactive compounds, which are present in an administered drug, should be delivered not only to a given tissue but precisely to selected cells and often even to particular intracellular compartments. In addition, they should be present there within a required concentration range for a given time. Such delivery assures the avoidance of undesired exposure of healthy tissues to bioactive compounds. Delivery of the bioactive substances to selected cells in chosen organs, which is commonly called targeted drug delivery, requires carriers with sizes close to or smaller than cell dimensions. Important constraints on properties of the polymer carrier particles are imposed by the chosen administration route.

In the case of the quite often used oral drug delivery, the carrier and its contents are exposed to very strong changes of pH, from strongly acidic pH = 1.5 in stomach, via slightly basic pH = 8.5 in duodenum to almost neutral pH ranging from 6.30–7.49 [1]. Moreover, beginning from the oral cavity, the drugs are exposed to contact with digestive enzymes. At such conditions, many biologically active substances cannot survive intact and should be protected by the carrier. Design of carriers should take into account their mobility in viscoelastic mucus toward mucosa lining the inner part of the small intestine and colon. Diffusion of particles in mucus strongly depends on their diameters and adhesive interactions with molecules constituting the mucus gel [2]. Nanoparticles (i.e., particles with equivalent diameters ranging from approximately 1–100 nm [3] which do not bind to mucus by physicochemical interactions diffuse by standard Brownian motion. Small microparticles (microparticles are defined as particles with equivalent diameters in the range from about 0.1–100 nm [3]) with diameter of the order of mucus gel pores (about 0.2 μm) which are chemically inert toward mucus experience significant hindrance from the gel mesh. Their movements are correlated with movements of mucus chains [2]. Movement of larger (>0.5 μm) microparticles which are chemically inert toward mucus depends not only on the local fluctuations of mucus chains but to a great extent on mucus movements in the larger scale. Thus, due to the hindered Brownian diffusion, the large microparticles may not have enough time to cross the mucus layer toward the epithelium due to the mucus clearance from the intestines.

The particles which successfully passed through the mucus barrier may either release their bioactive payload next to the epithelium or cross the epithelium barrier. Bioactive compounds released at the epithelium may cross the epithelium barrier in a similar manner to the digested food components and eventually reach the bloodstream. Such a delivery pathway is appropriate in the case of bioactive compounds, for which blood is the target (e.g., insulin controlling glucose level in the blood). Transfer of drug carriers through the endothelium is needed when the bioactive substance must be delivered predominantly to particular loci. Active transport of nano- and microparticles across the epithelium occurs mostly due to function of microfold cells (M-cells) and strongly depends on particle size and physicochemical properties of the particle interfacial layer.

Transdermal delivery of particles loaded with bioactive compounds poses serious problems due to the thickness and structure of skin constituting a less permeable barrier than mucosa. Thus, transdermal delivery of macromolecules is ineffective without physical support (e.g., microneedles, electroporation, electrophoresis, sonoporation, sonoporesis, microjets, or laser), often combined with chemical permeability enhancers [4,5]. This subject, however, is outside the scope of the present review and will not be discussed.

Pulmonary and nasal deliveries are very convenient and comfortable for patients [6,7,8,9]. In part, drug delivery mechanisms by these routes are similar to those of the oral route. Namely, the particles must cross the mucus and mucosa barriers before they reach the blood. However, due to the anatomy of the nasal cavity and lungs, particles with appropriately tailored properties are required. It is worth noting that nasal delivery attracts special attention because it contains the only site (olfactory region) where the central system nerves are exposed in the mucosa layer, creating some hope for bypassing the blood–brain barrier during drug delivery to the brain. The surface of the nasal cavity is approximately 160 cm^2^ (about 96 m^2^ taking into account microvilli). Unfortunately, the surface of the olfactory region is close to 5 cm^2^ (about 0.3 m^2^ taking into account microvilli) [8].

One of the common routes for drug administration is by injection. There are various kinds of injection procedures, which are chosen depending on drug physicochemical properties, drug functions, and target organs and tissues. The least invasive is subcutaneous injection, very often used for vaccination. The abundant presence of capillary lymph vessels (CLV) in skin and subcutaneous tissue facilitates drainage of vaccine containing nanoparticles and their effective transport to lymph nodes (LNs). An uptake of nanoparticles by antigen-presenting cells (APCs) starts a cascade of events directed toward development of immune response [10]. Drainage of nanoparticles targeted to LNs, time of their residence in these structures, as well as their uptake by APCs, final lysosomal escape, and cytosol delivery may depend on nanoparticle size, shape, rigidity, and chemical composition of their interfacial layer [10,11,12,13,14,15]. However, there are ambiguous opinions on the influence of the abovementioned parameters and properties of nanoparticles on the efficiency of immunization. Drug administration by intramuscular injection is very simple and opens interesting opportunities. Muscles are responsible for creation of force and induction of movement. To fulfill these functions they require an efficient supply of substances used as sources of energy, such as glucose and oxygen. Both these compounds are transported by blood and therefore muscles are strongly vascularized. Along with blood, side products are also removed from the muscles. The latter function makes muscles an excellent candidate for the depositing of bioactive substances, which when entering the blood can be transported over the organism. In the case of intramuscular drug administration there are two mechanisms of drug distribution. According to the first one, the bioactive substance is gradually released from the biodegradable carriers introduced into the muscle and then transported by blood to various organs and tissues. The carrier particles should be large enough (about 10 μm or more) to stay in the deposition site. This mechanism enables sustainable drug delivery during time controlled by microparticle degradation or drug diffusion from it [16,17,18,19,20,21]. The second mechanism requires small nanoparticles (with diameters usually lower than 100 nm), which together with their drug payload aggregate upon injection or when injected are suspended in a substance-forming gel in situ [22,23]. However, often it remains unclear whether in this case the drug is released from nanoparticles in the muscle or other organs, or whether drug-loaded nanoparticles are uptaken by blood and the drug is released later. One of the important routes of drug administration consists of intravenous injection. In this case the carriers containing the drug are instantaneously introduced into the blood stream and very quickly distributed in the whole organism. The ultimate localization of particle carriers depends on their specific interactions with cell membranes of particular organs and on the relationship between blood vessel diameters and diameters of drug carriers. The role of the first mentioned factor will be discussed in a more detailed manner in the later part of this review. The role of the second factor is simple. Organs with smaller diameters of capillary blood vessels are less accessible for the larger particle carriers. On the basis of literature data (see Reference [24]) it was noticed that particles with diameters exceeding 10 μm are too large to pass capillary blood vessels of the majority of organs and their action is not very far from the injection site. Particles with diameters in the range from 5–10 μm are preferentially captured in lungs; those with diameters below 5 μm are efficiently eliminated from the blood by the mononuclear phagocyte system. Particles with diameters ranging from 1–3 μm are captured in the spleen. Particles with diameters from 0.1–1 μm are captured in the liver and nanoparticles with diameters from 50–100 nm in bone marrow, respectively.

## 2. Polymers for Preparation of Drug Delivery Carriers

**Scheme 1:**
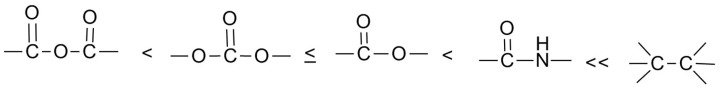
Order of resistance of chemical linkages toward hydrolysis.

When polymer carriers reach the targeted tissue and release the bioactive substance, the polymers should be eliminated from the organism. In turn, accumulation and prolonged presence of foreign polymer material may cause various unwanted side effects such as inflammation, clot and cyst formation, immune response, and others. In principle, there are three basic pathways for polymer clearance. The clearance of polymer nanoparticles with diameters ranging from six to several hundred nanometers proceeds effectively by the hepatobiliary route [25]. The nanoparticles brought to the liver with blood are endocythosed by hepatocytes, enter the bile duct via bile canaliculi, are delivered to the digestive tract, and are eventually eliminated with feces. The hepatobiliary clearance may require duration from a few hours to several weeks. For example, about 35% of nanocapsules prepared from poly-L-lysine hydrobromide (*M*_w_ 15,000~30,000 g/mol) and poly-L-glutamic acid (*M*_w_ 30,000~50,000 g/mol) were eliminated from mice within 24 h [26]. The second pathway is by renal route; the third one starts with polymer degradation. Polymers removed by the renal method should be water soluble and have an upper limit of molar mass of about 40,000 g/mol [27,28]. However, it is worth noting that it is not molar mass that matters in renal clearance but the size of polymer chains or nanoparticles; macromolecules with the same molar mass but a different chemical architecture may have different dimensions. Generally, for efficient glomural filtration, hydrodynamic diameters of polymer coils or nanoparticle size should not exceed 10 nm [29,30,31]. Degradation of polymers with molar masses larger than the abovementioned upper limit leads to polymers with shorter chains, which eventually may be small enough to be removed via kidney. It is possible also, depending on the degradation mechanism, for degradation to yield small molecules, which could be metabolized in the organism to CO_2_ and water. Rates of polymer degradation depend on their chemical structure, crystallinity, size and shape of degraded objects, temperature, and pH at which degradation takes place; however, the general order of resistance to degradation is like the one shown in Scheme 1. The least stable are carboxyanhydride groups, the most stable (with a few exceptions that are practically non-degradable) are C–C linkages. The exceptions are polycyanoacrylates, in which the presence of electron-withdrawing –C≡N groups and ester groups may facilitate degradation. The most important classes of synthetic and natural polymers used for preparation of drug carriers are presented below, beginning with the ones that are most susceptible to hydrolysis. It should be noted that copolymers and their modified derivatives, not pure homopolymers, are used for this purpose.

### 2.1. Polyanhydrides

**Scheme 2:**
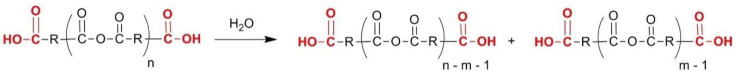
Degradation of polyanhydrides by hydrolysis.

The first reports on using polyanhydrides for fabrication of drug delivery systems were published in the mid-1980s of the previous century [32,33,34]. These early studies were mainly related to macroscopic drug-eluting implants. Hydrolytic erosion of these implants proceeds at the surface, decreasing molar masses of polymers and producing shorter chains with carboxylic acid groups at the ends (see Scheme 2). Obviously, acidic and basic conditions facilitate hydrolysis of polyanhydrides of carboxylic acids.

**Scheme 3:**
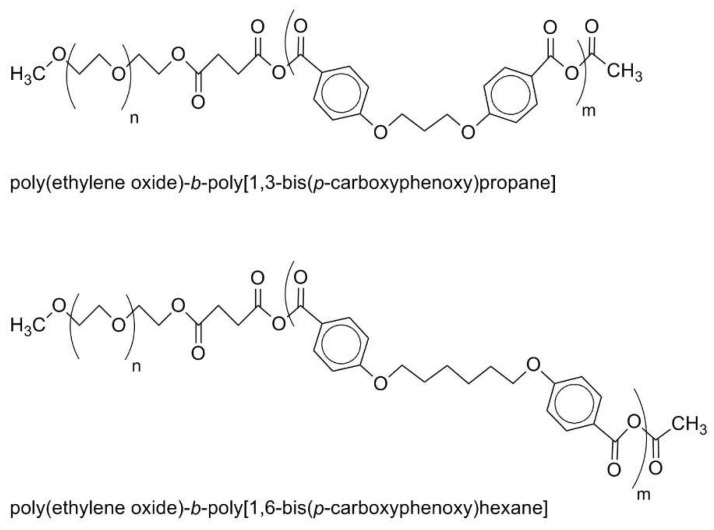
Amphiphilic copolymers with poly(ethylene oxide) and poly(1,3-bis(p-carboxyphenoxy)propane) or poly(1,6-bis(p-carboxyphenoxy) hexane) blocks.

Later, several kinds of polyanhydride nano- and microparticle drug carriers were developed [35,36,37,38,39,40,41,42,43,44,45]. Several of these studies were devoted to microspheres and microcapsules prepared from poly(sebacic anhydride) [38,39,44]. There studies also investigated microparticles produced from other polyanhydrides such as poly(bis(p-carboxy-phenoxy) propane) [35] and poly(fumaric anhydride) [36], copolymers of sebacic acid with carboxyphenoxypropane [35], 1,6-bis-p-carboxyphenoxy)hexane [40], and ricinoleic acid [42]. Polymeric micelles were produced by assembly of amphiphilic copolymers containing hydrophilic poly(ethylene oxide) and poly(1,3-bis(p-carboxyphenoxy)propane) or poly(1,6-bis(p-carboxyphenoxy) hexane) hydrophobic blocks (see Scheme 3) [41].

It is worth noting that polyanhydrides produced exclusively by polycondensation of diacids always bear carboxylic acid end-groups, which might be used for further polymer modifications. Even in syntheses, in which monoacids are used as molar mass regulators (see Scheme 3), the hydrolysis of controlled low fraction of anhydride linkages would produce carboxylic acid end-groups.

### 2.2. Polycarbonates

**Scheme 4:**
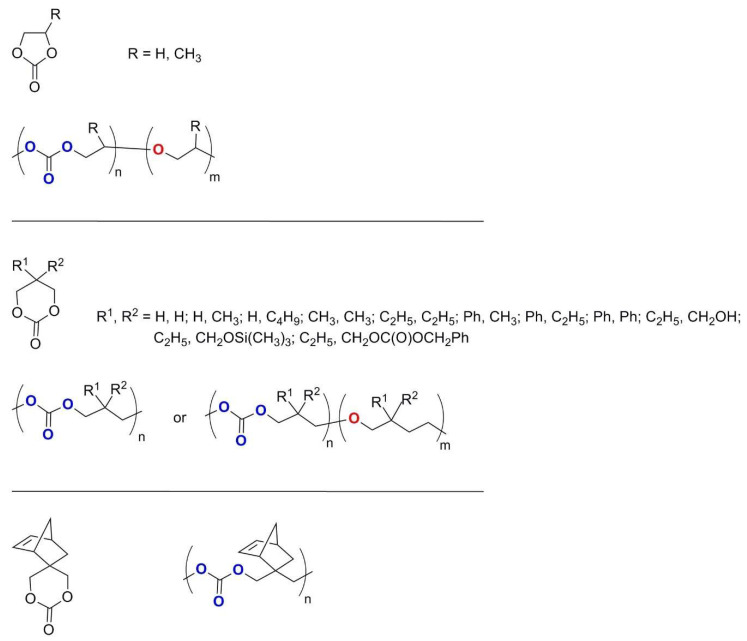
Structures of five- and six-membered carbonates and polymers obtained by their ROP processes (based on data from References [51,56]).

Polycarbonates most often used for medical applications are obtained by ring-opening (ROP) polymerization of ethylene carbonate (EC), trimethylene carbonate (TMC), and their substituted derivatives. Copolymers of these monomers were also used. The first reports on polymerization date to the early 1930’s of the previous century, when W.H. Carothers and W.J. Van Natta published results of their studies on polymerization of EC, TMC, and cyclic carbonates with larger rings [46,47]. Later, studies in this field enabled much better understanding of cyclic carbonate polymerization and degradation of synthesized polymers [48,49,50,51,52,53,54,55]. A comprehensive review on this subject was published by G. Rokicki [56]. Structures of ethylene carbonate, trimethylene carbonate, their substituted derivatives, and relevant polymers are shown in Scheme 4.

It is worth noting that depending on polymerization conditions, decarboxylation accompanies propagation, leading to release of CO_2_ and formation of some amount of ether linkages in polymer chains [57,58].

**Scheme 5:**
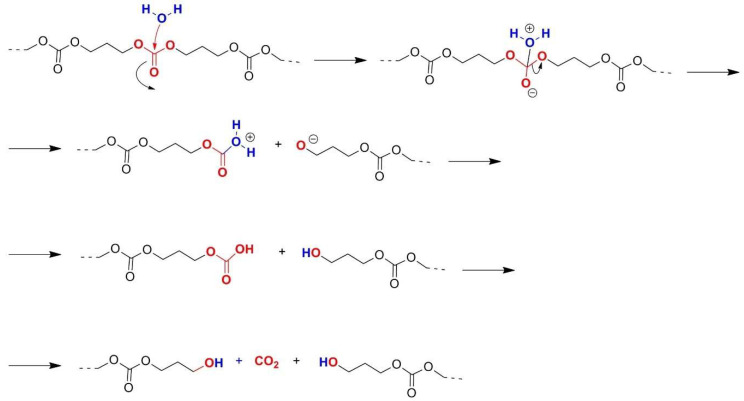
Hydrolysis of poly(trimethylene carbonate) (based on data from Reference [50]).

**Scheme 6:**
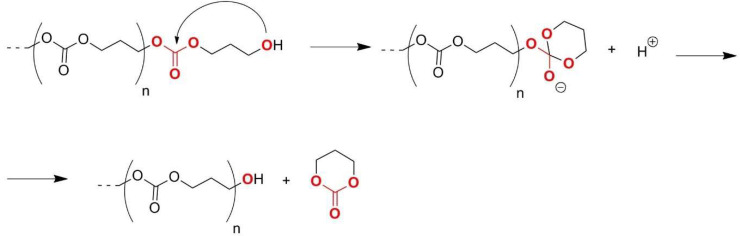
Degradation of poly(trimethylene carbonate) by depolymerization (based on data from Reference [56]).

Degradation of polycarbonates consists of hydrolysis of carbonate linkages with concomitant release of CO_2_ and subsequent depolymerization involving –CH_2_OH and adjacent –OC(O)O– groups (see Scheme 5 and Scheme 6) [50,54].

Scheme 5 and Scheme 6 show that degradation of poly(trimethylene carbonate) leads to production of short chain oligo(trimethylene carbonate)s with –OH end groups, CO_2_, and monomer. Any further hydrolysis of trimethylene carbonate yields CO_2_ and 1,3-propane diol. Thus, products of degradation of polycarbonates are free from any acidic groups, which when in contact with tissue may cause irritation or inflammation.

Literature from the last ten years contains many examples of drug carriers prepared from polycarbonates and polycarbonate-containing copolymers [59,60,61,62,63,64,65,66,67,68,69,70,71,72,73,74,75,76,77,78,79,80,81].

### 2.3. Aliphatic Polyesters

**Scheme 7:**
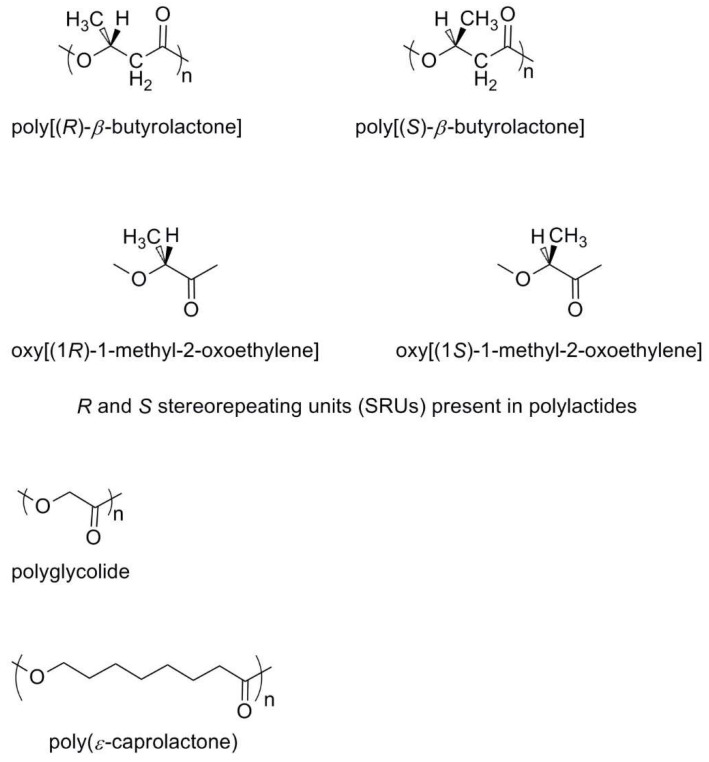
Structure units of polyesters commonly used for preparation of drug nanocarriers. Terminology of polylactide structure units is based on IUPAC recommendations [88].

Aliphatic polyesters constitute a class of synthetic polymers most often used for preparation of degradable nano- and microcarriers of bioactive compounds. The polymers are usually synthesized by ring-opening polymerization (ROP) of cyclic esters. An exception is poly(*β*-butyrolactone), which could also be produced by bacteria in biotechnological processes [82,83,84] or by copolymerization of propylene oxide and carbon monoxide [85,86,87]. Synthesis of this polymer in bacteria was developed on an industrial scale first by ICI and Monsanto. Activity in this field has been continued by other companies. Structures of polyesters most often used for preparation of drug carriers are shown in Scheme 7.

It is worth noting that poly(*β*-butyrolactones) and polylactides contain chiral carbon atoms in the main chains. Distribution of stereospecific units along the chains (polymer microstructure) strongly affects polymer crystallization and eventually their hydrolytic degradation. Water cannot penetrate crystalline phase; thus, polymer crystals are degraded at the surface. In contrast, the diffusion of water into amorphous phase results in bulk degradation. However, in both cases shorter chains equipped at one end with hydroxyl and at the other one with carboxyl groups are produced.

Properties of aliphatic polyesters are tailored by using, instead of homopolymers, copolymers of basic monomers. Only in the last two years, many kinds of aliphatic homo and copolymers have been used for fabrication of nano and microcarriers of bioactive substances. The microcarriers were prepared in the form of nanoparticles [89,90,91,92,93,94], polymeric micelles [95,96,97,98,99,100,101,102,103,104], and nanocapsules [105].

### 2.4. Polyorthoesters

**Scheme 8:**
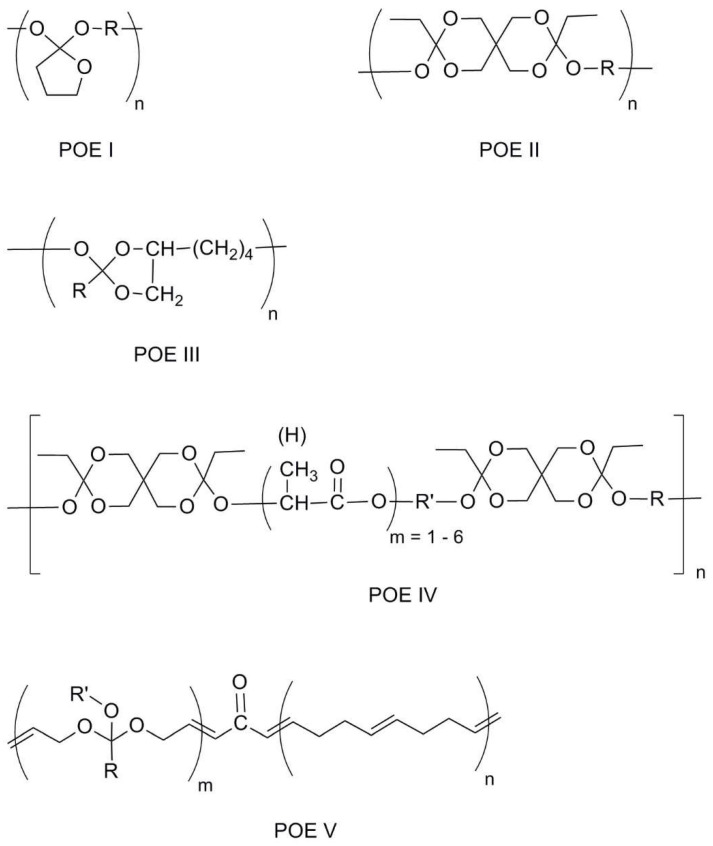
Structures of five major classes of polyorthoesters (POE) for medical applications [106,107,108].

Polyorthoesters are polymers containing, in the main chain, orthoester groups, which formally could be considered as esterification products of orthocarboxylic acid. Examples of polyorthoesters are shown in Scheme 8.

**Scheme 9:**
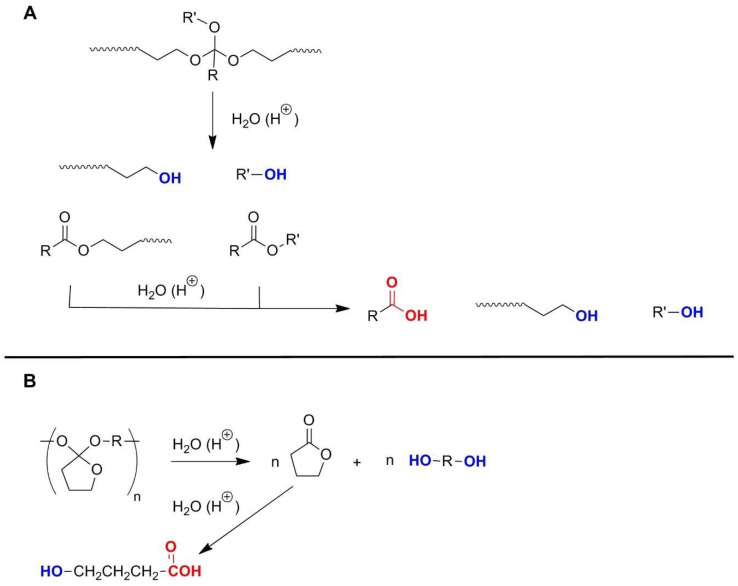
Schematic illustration of hydrolysis of polyorthoesters (**A**)—Class POE V, (**B**)—Class POE I.

Polyorthoesters are hydrophobic [106,107,108]. Water penetrates into polyorthoesters with difficulty; therefore, their degradation usually occurs at the surface. Products of hydrolysis contain mono-, di-, and multifunctional alcohols and carboxylic acid groups. Their structures depend on the chemical structure of the particular polyorthoester. Examples are shown in Scheme 9.

Hydrolysis of polyorthoesters proceeds in two stages [107,108]. In the first one, polymers with electrically uncharged alcohol and ester end groups and (in the case of POE I) γ-butyrolactone are formed. These species could be eliminated via renal routes. In the second stage, the subsequent hydrolysis of the remaining γ-butyrolactone yields low molecular weight compounds with carboxylic acid groups.

In 2004, J. Heller indicated the possibility of using polyorthoesters for fabrication of drug carriers by microencapsulation methods [109]. Later Ch. Wang et al. elaborated the polyorthoester-based microspheres for DNA vaccine delivery [110].

### 2.5. Polyalkylcyanoacrylates

**Scheme 10:**
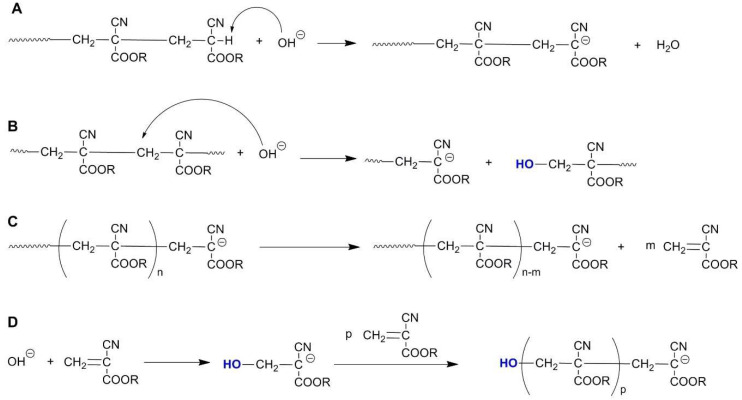
Degradation and chain reshuffling and repolymerization: (**A**)—activation of chain end-groups by proton transfer, (**B**)—formation of chains with active end-groups by chain scission, (**C**)—depolymerization by unzipping of chains with anionic end-groups, (**D**)—repolymerization of alkylcyanoacrylates initiated with hydroxide anions.

Polyalkylcyanoacrylates (PCA) are unique as materials for production of drug carriers. In spite of the fact that main chains of PCAs contain exclusively carbon–carbon bonds, these polymers may undergo degradation by unzipping depolymerization and parallel repolymerization with formation of some new chains (see Scheme 10) [111]. It should be noted that because chain scission and reshuffling by unzipping and repolymerization yield polymers with the same main-chain and end-group structures, determination of contribution of these processes to chain length redistribution is not easy. Nevertheless, because chlorine end-capped poly(butylcyanoacrylate) is resistant to chain reshuffling by unzipping and repolymerization, at least for this polymer, formation of chains with reactive anionic chain-ends by chain scission does not seem very important [111]. It should also be noted that the side ester groups in PCAs are prone to enzymatic hydrolysis catalyzed by esterases [112,113,114]. This results in the formation of water-soluble polyanionic polymers. Provided that their molar masses are close to 40,000 g/mol or lower, the polymers could be eliminated from the human organism via the kidney.

**Scheme 11:**
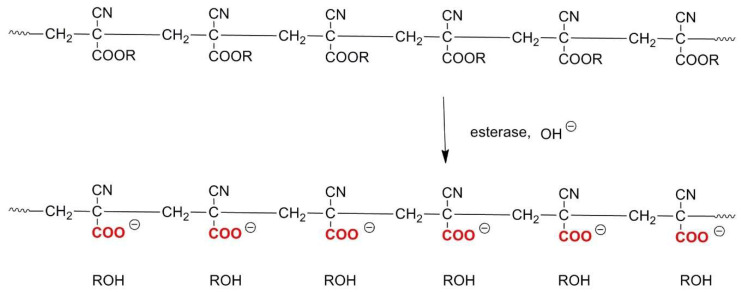
Enzymatic hydrolysis of ester groups in poly(alkylcyanoacrylates).

Scheme 10 and Scheme 11 illustrate degradation of PCAs [111,112,113,114]. According to existing opinion, the degradation by hydrolysis of ester side groups in vivo plays the predominant role [115].

Poly(alkylcyanoacrylate) nanoparticles and nanoparticles containing copolymers with poly(alkylcyanoacrylate) blocks were used as carriers of bioactive substances. Here we cite only some original papers published during the last ten years [116,117,118,119,120] and some comprehensive reviews covering this subject [121,122,123,124,125,126].

### 2.6. Biopolymers

Biopolymers comprise peptides and proteins, oligo- and polysaccharides, as well as oligo- and nucleic acids and polynucleotides [127]. From these biopolymers only peptides and proteins and oligo- and polysaccharides are used for fabrication of drug nano- and microcarriers. Nucleic acids from well-defined living organisms not only are too expensive to be used on a large scale for drug encapsulation but, what is even more important, when introduced into human tissue may cause some unwanted side effects. Proteins should also be used with care for preparation of drug carriers (they may cause undesired immune reactions). However, several of them, such as human serum albumin, silk protein fibroin, gelatin, legumin, gliadin, 30Kc19, ferritin, and lipoprotein listed in the recent review were used for preparation of nanocarriers of bioactive compounds [128]. Depending on chemical structure, proteins contain various functional groups: amine (in arginine, lysine, and histidine residues), carboxyl (in aspartate and glutamate residues), carboxyamide (in asparagine and glutamine residues), hydroxyl (in serine and threonine residues), and thiol (in cysteine residues). It should also be noted that proteins often contain various functional structure elements created by the specific arrangement of protein chain fragments. A typical example is human serum albumin used to complex drugs and other bioactive compounds [129,130,131]. Special attention attracted derivatives of relatively short polypeptides, particularly block copolymers containing blocks of poly(ethylene oxide) and poly(aspartic acid); carboxyl groups of poly(aspartic acid) could easily be modified and/or used for drug conjugation. Depending on block length, they self-assemble into polymeric micelles. In spite of the fact that the first paper on the aforementioned copolymers as drug carriers was published by Kataoka et al. more than thirty years ago [132,133], studies in this field have been developing especially fast in the last few years [134,135,136,137].

**Scheme 12:**
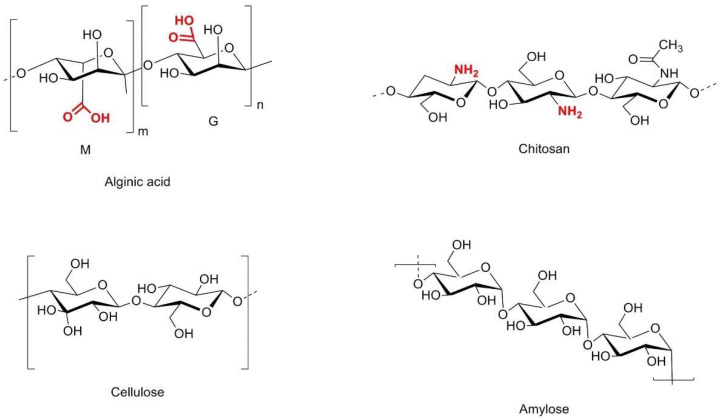
Structures of natural polysaccharides commonly used for fabrication of drug carriers.

Polysaccharides and their derivatives constitute another important group of functional biopolymer-related polymers used for fabrication of nano- and microcarriers of bioactive compounds. In addition to hydroxyl groups, which are present in all polysaccharides, some of them also contain carboxylic (e.g., alginate) or amine (chitosan) groups. Examples of chemical structures of polysaccharides often used in drug delivery systems [138] are shown in Scheme 12. These examples include: alginic acid composed of blocks of (1→4)-linked β-d-mannuronate (M) and α-l-guluronate (G) residues, chitosan containing randomly distributed β-(1→4)-linked d-glucosamine and *N*-acetyl-d-glucosamine residues (polysaccharide produced by partial deacetylation of chitin), cellulose containing β(1→4) linked d-glucose residues and amylose—an important component of starch—composed of α-d-glucose residues, bonded through α(1→4) glycosidic bonds.

The list of polysaccharides used for preparation of drug carriers is continuously growing. Today it also includes hyaluronic acid [139], xanthan gum [140], and additionally: pectin, carrageenan, carboxymethyl cellulose, and dextran [138,141]. Some of them contain sulfate anionic groups, such as chondroitin sulfate, carrageenan, porphyran, fucoidan, and ulvan [141,142,143]. However, one should note that the hydrophilic/hydrophobic balance of polysaccharides is controlled by modifications introducing hydrophobic groups [144].

Proteins and polysaccharides are quite resistant to hydrolysis at pH close to neutral. Nevertheless, one should note that some of them (chitosan and its acrylated derivatives) indicate certain instability of physical parameters during storage even of lyophilized dry samples [145,146]. At low pH or by enzymatic catalysis (for dextran at pH 1.4 and 1.8 or using dextranase), hydrolysis provides lower molar mass water-soluble samples suitable for preparation of drug delivery formulations [147].

Degradation of proteins occurs mainly by enzymatic pathways, regardless of whether in vitro [148] or in vivo [149,150]. Orally administered protein carriers are degraded by digestive enzymes [149] whereas those introduced directly into the bloodstream enter cells by endocytosis and undergo lysosomal degradation [142].

## 3. Preparation of Functionalized Nano- and Microparticles

There are two major strategies for the manufacturing of functionalized nano and microparticles. The first one, based on dispersion and emulsion polymerization methods, enables preparation of particles directly from monomers. The second one is based on physical processes of self-assembly of homo and/or copolymer molecules or on special particle-forming techniques (e.g., spray drying and microfluidics). The first strategy is used very rarely for preparation of functional nano and microparticle drug carriers. We discuss it very briefly for the sake of completeness only. The vast majority of nano and microparticle drug carriers are prepared according to the second very versatile strategy enabling the use of polymers with tailored chemical and physical properties.

### 3.1. Functional Nano- and Microparticles Prepared by Polymerization

The first papers on the direct synthesis of functionalized nano and microparticles were published in 1994. P. Teyssié et al. reported on synthesis of polyester nanoparticles by coordination copolymerization of ε-caprolactone and glycolide initiated with ω-Al-alkoxide-poly(ε-caprolactone) macroinitiator [151]. Synthesis was carried out in THF solvent, in which the THF soluble poly(*ε*-caprolactone) blocks ensured needed colloidal stability of nanoparticle suspension. Slomkowski et al. described synthesis of poly(*rac*-lactide) and poly(*ε*-caprolactone) nano and microparticles by ring-opening dispersion polymerization of parent monomers. The polymerization was initiated with Et_2_AlOEt_2_ and carried out in 1,4-dioxane/heptane mixed solvents [152]. Colloidal stability of the particles was assured by addition of poly(dodecyl acrylate)-*g*-poly(ε-caprolactone) stabilizer. Number average diameter (*D*_n_) and diameter dispersity factor (*Đ*_D_ = *D*_w_/*D*_n_, where *D*_w_ denotes weight average diameter) of poly(ε-caprolactone) particles were 0.63 μm and 1.038, respectively. For poly(*rac*-lactide) the abovementioned parameters were *D*_n_ = 2.50 μm and *Đ*_D_ = 1.15. The synthesized particles were functionalized by coating them with proteins (human serum albumin and γ-globulin). Later studies revealed that better tailoring of poly(dodecyl acrylate)-*g*-poly(ε-caprolactone) structure (using the stabilizer with a ratio of *M*_n_ of poly(ε-caprolactone) grafts and *M*_n_ of the whole copolymer equal to 0.25) enabled synthesis of poly(*rac*-lactide) microspheres with *Đ*_D_ = 1.03 [153].

Synthesized polyester particles were built of macromolecules with only terminal reactive hydroxyl end-groups. Therefore, for high molar mass polymers, the concentration of hydroxyl groups in the particles’ interfacial layer is quite low, too low for many applications. This inconvenience was eliminated by controlled hydrolysis of the particles’ interfacial layer, yielding new shorter chains with hydroxyl groups at one end and carboxyl groups at the other end [154]. The hydrolysis was carried out for a controlled time in ethanol containing a controlled amount of KOH and nonionic (Triton X-405) or ionic (sodium dodecyl sulfate—SDS, ammonium sulfobetaine-2—ASB) surfactants. Average diameters of particles decreased during hydrolysis by not more than 5%. The particles were colloidally stable in water. Presence of carboxyl groups in the interfacial layer enabled functionalization of the particles with 6-aminoquinoline making their surfaces fluorescent.

### 3.2. Nano- and Microparticles by Self-Assembly of Functional (Co)Polymers

Nano- and microparticles were obtained in various precipitation processes from premade amphiphilic block copolymers or homopolymers. The biomedical applications impose several restrictions on particles, including colloidal stability in aqueous media. Thus, the hydrophobic blocks (or polymer segments) forming a core of particles should be surrounded by hydrophilic corona.

Nano- and microparticles could be obtained by various techniques yielding particles with various sizes, size distribution, and morphology.

Usually, the process consists of dissolving amphiphilic block copolymer in organic solvent or a mixture of solvents (in order to facilitate its solubility) and transfer of the copolymer solution to an aqueous medium. The final particles’ architecture depends on the method of addition, temperature, time of mixing, and interactions of polymer solvent and non-solvent (e.g., their miscibility).

The particles were usually obtained by one of the following techniques [155,156,157,158,159]:

- Nanoprecipitation covering “classical” nanoprecipitation and “reverse” nanoprecipitation;
- Flash nanoprecipitation;
- Solvent evaporation/dialysis;
- Salting out;
- Miscellaneous methods including spray-drying.

Classical nanoprecipitation consists of a slow (dropwise) addition of solution containing polymer dissolved in an organic medium to continuously stirred non-solvent, which is miscible with solvent. The process results in fast mixing of solvent and non-solvent. This results in polymer or copolymer self-assembly, yielding particles. Classical nanoprecipitation usually produced micelles. In the “reverse” process the non-solvent is added dropwise to the stirred copolymer solution. Nanoprecipitation sometimes produces nano- or microparticles with complex bicontinuous morphology of hydrophilic and hydrophobic nanophases.

In flash nanoprecipitation, the polymer solution and non-solvent are introduced (injected) from separate containers, with a controlled velocity, to a mixing chamber, within ca. 1 millisecond. In flash nanoprecipitation, contrary to classical nanoprecipitation, the uniform nucleation and growth of particles takes place. In consequence, the process yields particles with vesicular or bicontinuous architecture [157]. The final particle morphology depends on copolymer concentration and solubility of individual blocks of copolymer in polymer solvent and non-solvent.

Nano- and microparticles were also obtained by dialysis of copolymer solution against non-solvent miscible with solvent. During this process, solvent is slowly replaced by non-solvent. Typically, the ratio of solvent and non-solvent volume is significantly lower than 1 [158].

The alternative to the aforementioned precipitation methods is salting-out. In this procedure, the polymer and drug solution in organic solvent miscible with water is emulsified in water containing a high concentration of salting-out agents such as sodium or magnesium chloride, sucrose, and often surfactant. The emulsion is added to a large volume of water and formed particles are purified by cross-filtration. The method is useful for preparation of large amounts of drug-loaded nanospheres, but the product requires removal of salts, which is tedious and time consuming. Another disadvantage of the salting-out method consists of incompatibility of salts with biologically active compounds [155].

Preparation of particles by the spray-drying method consists of atomizing polymer solution or dispersion in hot-air flow. The method, being very fast and efficient, is suitable for manufacturing particles on an industrial scale. Unfortunately, control of diameters and diameter dispersity for particles prepared by spray-drying are not fully controlled. Moreover, temperature of the nozzle of the spray-dryer is usually maintained close to 100 °C, which poses a problem for thermally unstable drugs.

The majority of the aforementioned methods require organic solvents for preparation of the polymer solution. Traces of these solvents may be harmful when they remain in drug-loaded particles. A few years ago, a new method was developed for fabrication of poly(lactide-*co*-glycolide) drug carriers without using typical polymer solvents [159]. The method consists of three steps. In the first one, poly(lactide-*co*-glycolide) and the drug were dispersed by homogenization in triacetin (1,2,3-triacetoxypropane) containing poly(ethylene oxide) oligomers or Tween used as surfactants. This step yielded so called “embryonic microspheres”. The primary dispersion was added dropwise to continuously homogenized aqueous surfactant solution. The process produced microglobules dispersed in a continuous phase. When the dispersion was injected into the buffer, the microglobules hardened to solid particles. The drug release profile from these particles was predictable and well controlled.

There are reports on preparation of nanoparticles in processes based on supercritical carbon dioxide techniques, which facilitate removal of traces of organic solvents from the product [160,161]. These processes are promising; however, they still require improvements to assure better control of particle diameters and diameter distribution.

**Figure 1:**
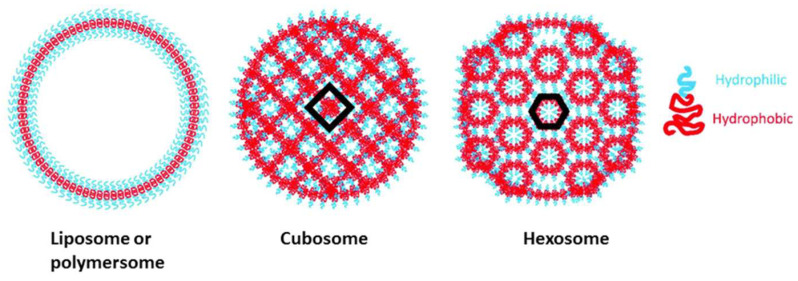
Arrangements of amphiphilic block copolymers in liposome (or polymersome), cubosome, and hexosome type particles. Particle morphology depends on symmetry/asymmetry of hydrophilic/hydrophobic parts [156].

Morphology of nano- or microparticles obtained by self-assembly of macromolecules depended on techniques chosen for their fabrication and was strongly affected by amphiphilic copolymer architecture. Topology and length of hydrophilic and hydrophobic blocks were very important. The amphiphilic copolymers self-assembled into liposome-like polymersomes, polymeric micelles, and microglobules composed of the polymer micelles organized into more complex structures, e.g., cubosomes or hexosomes, schematically shown in Figure 1.

It is worth noting that particles with bicontinuous mesophases were formed by self-assembly of linear di-, triblocks, combs, or multi-arm macromolecules. Morphology of particles was induced by reduction of free energy by stretching the hydrophobic blocks in polymersome bilayers or in cores of polymer micelles, interfacial tension between the core and solvent, and repulsion of chains in hydrophilic corona.

The packing parameter *P*, describing the shape of individual macromolecules of amphiphiles, is defined as follows in Equation (1):

*P = V/a_o_l_c_* (1)

where *V* denotes volume of hydrophilic part, *a_o_* surface area covered by hydrophilic part at hydrophilic–hydrophobic interface, and *l_c_* critical length of the hydrophobic segment (minimal length required for amphiphilic copolymers to self-assemble) [162,163].

It was noticed that with increased values of packing parameter the order of structures formed by self-assembly of amphiphilic macromolecules was as follows: spheres, cylinders, or triply periodic minimal surfaces, vesicles or lamellar structures, and corresponding inverted structures.

Amphiphilic blocks’ ratio parameter (*f*), defined as the ratio of molar masses of hydrophilic and hydrophobic components of amphiphiles, was also used for prediction of mesophase morphology in formed structures [163].

Authors of many papers reported on relations between the fraction of hydrophobic blocks in linear amphiphilic diblock and triblock copolymers and formation of internal bicontinuous mesophases in particles. For instance, bicontinuous internal architecture of cubosome or hexosome type was obtained for polystyrene-*b*-poly(ethylene oxide) (PS-*b*-PEO) for *f* in the range 0.057–0.091 [164]. Self-assembling of dendritic copolymers with three PEG branches (*b*PEG-PS) with *f* in the range 0.054–0.197 yielded various bicontinuous cubic microstructures. In particular, bicontinuous cubic structures were obtained for *b*PEG–PS with PEG branches (*b*PEG) of M_n_ = 550 g/mol and for *f* parameter ranging from 0.071–0.078. However, the authors noticed that for *f* close to 0.05, the *b*PEG–PS copolymer with M_n_ = 750 g/mol of PEG branches, the cubic structures with varied symmetries were created. Other linear di- or triblock copolymers containing PS as the hydrophobic component in their composition have been described.

Today, attention is more often focused on particles with non-spherical shape. However, the role of particles’ shape in particle–cell interactions is still far from being sufficiently well understood.

Synthetic routes for preparation of non-spherical nano- and microparticles depend on many parameters, including interfacial properties of the emulsion droplets, size of particles, separation of individual polymer segments within the copolymer droplet, and evaporation rate of the solvent in which the copolymer was dissolved [165]. The detailed discussion of non-spherical particle formation was presented in Reference [165]. It is worth noting that knowledge of preparation and degradation of biodegradable non-spherical particles is almost non-existent.

For targeted drug delivery, often nano- and microparticles are needed, which could be preferentially bonded to particular cell types. Such particles should have appropriate reactive groups able to react with selected cell receptors. However, very often shells of nano- or microparticles do not contain chemical groups needed for further immobilization of biologically active species. These surface reactive groups could be introduced during particles’ synthesis by the addition of reactive surfactants, usually in amounts below 10% of the monomer concentration or, less often, by using monomers containing chemical species needed for further particle functionalization. The biological molecules used in particles’ synthesis often contain amine, hydroxyl, carboxyl, thiol, guanidine, or imidazol groups [166]. These functional groups on the particles’ surface enable the binding of biologically active compounds specifically interacting with cell receptors.

The particles’ core is very often crosslinked via reversible bonding, which disrupts under influence of pH change (usually pH in tumor tissues is acidic) or redox agents.

Table 1 contains representative examples of amphiphilic copolymers obtained via synthetic routes (pos.1–24) and natural polymers or their chemical modifications (pos. 25–31) with or without functional groups; compounds used for preparation of nanoparticles.

Chains of many types of amphiphilic copolymers contained functional group(s) at one or both ends. These end-groups were usually formed by deactivation of propagating species after completion of the polymerization. However, for many applications, copolymers containing a much larger number of functional groups were needed. Such copolymers were obtained using monomers with protected functional groups. After copolymer synthesis and deprotection of functional groups, the final products were obtained. For example, many copolymers with functionalized polycarbonate blocks were synthesized in this way (see Table 1, pos. 7–24). When functional groups were not reactive toward propagating species, their protection before copolymerization was not needed (see Table 1, pos. 7–10, 15, 17). Conjugation of synthetic polymers with natural poly and oligomers, such as poly- and oligosaccharides, proteins, peptides, poly- and oligonucleic acids, introduced such reactive groups as –COOH, –NH_2_, –OH, –NH_2_, –PO_4_^2−^, –N(CH_3_)_3_ (Table 1, Refs. [181,182,183,184,185,186,187,188,189,190,191,192,193,195,197,199,212]).

The aforementioned functional groups, when present in nanoparticles’ interfacial layer, could modify nanoparticle–cell interactions and may be used for binding biomolecules, specifically recognizing particular cells.

**Scheme 13:**
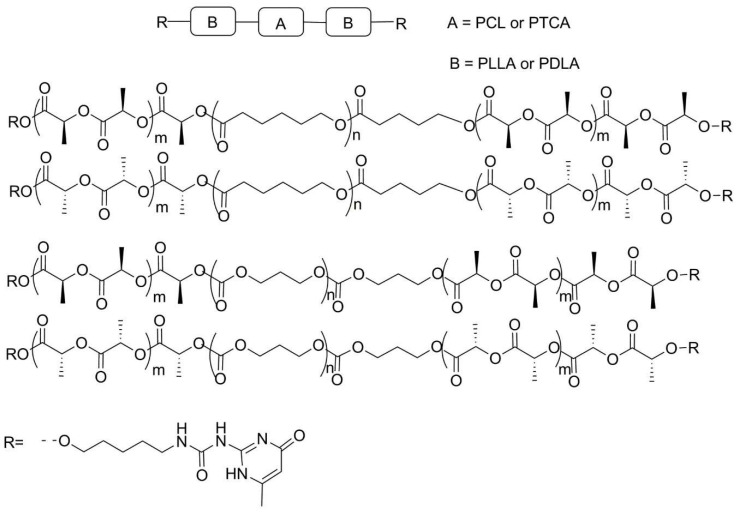
Structure of bifunctional triblock polyester copolymers used for formation of nano- and microparticles.

In some instances, whole copolymer blocks could be treated as “functional groups”. For example, triblock copolymers composed of poly(l- and poly(d-lactide) side blocks functionalized with 2-ureido-4-[1H]-pyrimidinone at both ends and central poly(trimethylene carbonate) or poly(ε-caprolactone) block (see Scheme 13) were able to self-assemble with formation of stereocomplexes of poly(d-lactide) and poly(l-lactide) into particles with diameters ranging from 0.1–10 μm. In these copolymers, poly(d-lactide) and poly(l-lactide) blocks could be treated as macro “functional groups” [169].

**Scheme 14:**
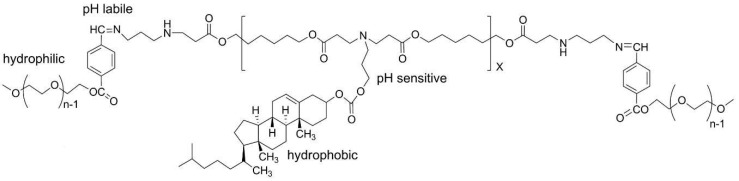
Structure of triblock copolymer containing poly(ethylene oxide) side blocks linked to poly(β-amino ester) with cholesterol labels via the pH sensitive linkers.

Functional groups could also be placed in precisely defined places on the copolymer main chain. Scheme 14 shows a triblock copolymer containing poly(ethylene glycol) side blocks linked to the central hydrophobic poly(β-amino ester) bearing cholesterol labels via the pH sensitive benzoylimine moieties [172]. The cholesterol groups were chosen to increase hydrophobicity of the central block and biocompatibility of the copolymer.

Self-assembly of copolymer chains yielded nanoparticles with poly(ethylene glycol) shells, preventing their rapid elimination from the organism. Upon uptake by cells and entry into lysosomes, the benzoylimine bonds are rapidly hydrolyzed and protecting poly(ethylene glycol) shells are removed from the particles. As a result, the nanoparticles free from the poly(ethylene glycol) coating can easily enter the cell nuclei.

The copolymers listed in Table 1 were used for preparation of functional nanoparticles by nanoprecipitation [39,76,80,81,171,174,207,210], reverse nanoprecipitation [78,79,167,169,179,209,211], solvent evaporation/dialysis [60,61,63,64,66,70,71,72,73,74,75,168,170,171,172,178,184,185,194], salting-out [189], freeze-drying [180,186], electrospraying [192,206], heat-denaturation [190,196,198], and ionic gelation [197]. The nanoparticles were loaded with doxorubicin [60,64,66,71,72,74,78,79,80,81,172,175,210,211], rhodamine B, *p*-nitroaniline, and piroxicam [172], gemcitabine [174], Nile red [72,73], paclitaxel [63,70,175,184,208], tacrolimus [70], temoporfin [76], bortezomib [75], platinum [176], amphotericin B [178], sulfadiazine [61], prednisone acetate and tegafur [180], carbazole [185], 10-hydroxycamptothecin [189], insulin [191], vaccines, genes [191], L-leucine [192], levofloxacin [196], calcitonin [197], genes [198,199,201], apolipophorin-III [200], imatinib [206].

Nanoparticles formed by the self-assembling of some polymers (often polysaccharides) are unstable and disintegrate under the influence of slight changes in their environment. Stabilization of such particles was achieved by crosslinking their cores, shells, or both. For crosslinking of nanoparticles, a method of alkene and thiol group coupling known as thiol-ene Michael addition was elaborated. The method was originally used for binding biological molecules, such as oligopeptides, proteins, and nucleic acids carrying sulfhydryl groups to nanoparticles with alkene moieties [211].

A similar procedure was applied for stabilization of the shell of nanoparticles formed by the layer-by-layer coating with alternated polyanionic and polycationic polymers. For example, silica nanoparticles were coated with alternate multilayers of poly(methacrylic acid) (PMA) and poly(vinyl pyrrolidone) (PVP) [213]. Each layer of PMA contained –SH or alkene groups. After assembly of the nine polymer layers, the particles were irradiated with UV light at 256 nm for 2 h, yielding crosslinks between PMA layers via thiol-ene coupling. In the subsequent step, the particles were PEGylated, using the reaction of PEG oligomer with maleimide end-groups and unreacted thiol-containing PMA (PMASH). Thiolated PMA was prepared in the reaction of cystamine dihydrochloride and PMA. The reaction was activated with *N*-(3-dimethylaminopropyl)-*N*’-ethylcarbodiimide (EDC). The free thiol groups of PMA were obtained by reduction of disulfide bridges with 1 M dithiothreitol (DTT) at pH = 8 [213]. The ene functionalized PMA was obtained in the reaction of 2-aminoethyl methacrylate hydrochloride (AEMH) and *N*-hydroxysuccinimide (NHS) [213].

**Scheme 15:**
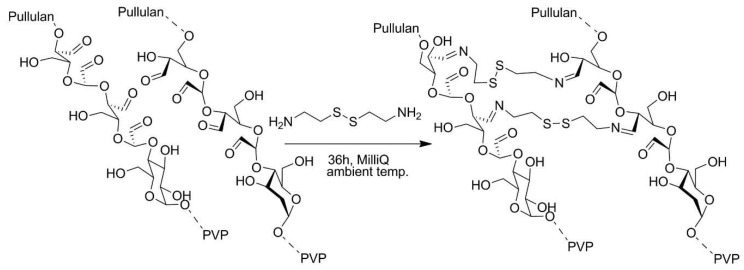
Stabilization of microparticles from Pull-*b*-PVP copolymer with oxidized hydroxyl groups by crosslinking using cystamine dihydrochloride.

The reversibly crosslinked hydrophilic particles were obtained by self-assembly of pullulan-*b*-poly(vinyl pyrrolidone) (Pull-*b*-PVP) copolymer [207]. Spherical particles with bimodal diameter distribution (1 and 150 nm) were formed from Pull124-*b*-PVP263. The particles were subsequently stabilized by oxidation of hydroxyl groups of pullulan to aldehyde groups (with sodium (meta)periodate) followed by crosslinking of aldehyde groups of neighboring chains by cystamine dihydrochloride (see Scheme 15). The particles could be disintegrated by redox cleavage of sulfur bridge using tricarboxyethyl phosphine (TCEP) at pH = 8 at 40 °C.

**Scheme 16:**
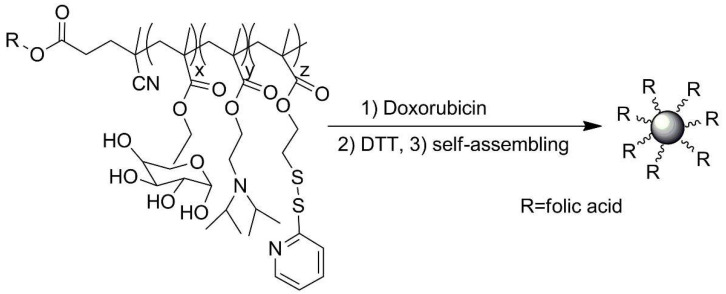
Schematic illustration of formation of nanoparticles loaded with doxorubicin by self-assembly of FA-PMgDP copolymer. Explanations are in text.

Using a similar approach, multifunctional core-shell particles with crosslinked cores were prepared from amphiphilic folic acid-poly(6-O-methacryloyl-d-galactopyranose)-*b*-poly(2-(diisopropylamino) ethyl methacrylate-co-pyridyl disulfide methylacrylate) (FA-PMAgGP-*b*-P(DPA-co-PDEMA)) denoted as FA-PM-*g*-DP block GAL-based copolymers. The poly(2-(diisopropylamino) ethylmethacrylate (PDPA) units were pH-sensitive (pKa of homopolymer was 6.3), whereas the poly(pyridyl disulfide methylacrylate) (PDEMA) units with reversible crosslinks were pH/redox-responsive [209]. Formation of nanoparticles as the result of self-assembling and self-crosslinking of the FA-PMgDP copolymer is shown in Scheme 16.

Nanoparticles produced by self-assembly of alginate grafted with poly(ethylene glycol methacrylate) alginate-(POEGMA) copolymers, facilitated by hydrogen bond formation between carboxyl groups of alginate and ether groups of poly(ethylene glycol), were reversibly crosslinked using CaCl_2_. The divalent Ca^+2^ cations, when interacting with two carboxyl anions of alginate, functioned as weakly crosslinking species. Nanoparticles loaded with doxorubicin and paclitaxel were obtained using the aforementioned method [208].

### 3.3. Hybrid Inorganic and Organic Nano- and Microparticles by Multistep Functionalization of Parent Particles 

There are many types of inorganic (e.g., gold, magnetite, silica) and polymer nano- and microparticles that in their original form do not contain functional groups. The groups, which are needed for particles’ particular application as drug carriers with enhanced circulation in the blood stream, provide particle delivery to targeted cells. There are two major strategies for functionalization of the aforementioned particles. The first consists of grafting, on the particle’s surface, low molar mass compounds containing required functional groups. The second one consists of irreversible coating of the pristine particles with synthetic or natural macromolecular compounds containing the needed functional moieties. Often the modifications are multistep processes. In this subsection, examples of such modifications are described.

Silica or silica-coated particles with Si-OH groups on their surface were grafted with trialkoxysilanes with amine groups [214,215] and polymers containing trihydroxysilane moieties and clickable alkyne functions [216]. The particles with amine functions were further modified with 2-bromoisobutyryl bromide, yielding particles with ATRP initiator and/or with lanthanide-doped 4-cyano-4-((dodecylsulfanylthiocarbonyl) sulfanyl) pentanoic acid-produced particles with chain-transfer agent used in RAFT polymerizations.

**Scheme 17:**
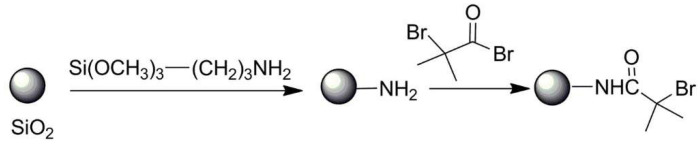
Reactions yielding silica particles with bromine containing ATRP initiating groups.

**Scheme 18:**
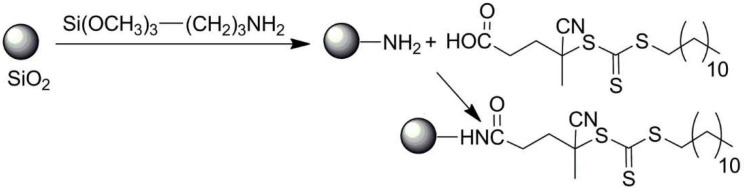
Reactions involved in a process leading to silica particles with RAFT chain transfer groups.

**Scheme 19:**
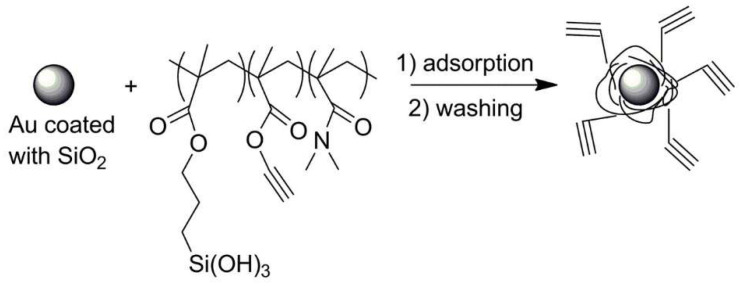
Reactions involved in a process used for production of gold coated with silica nanoparticles with polymer shells containing alkyne groups.

The relevant modification processes used for production of particles bearing ATRP initiators, RAFT transfer agents, and alkyne groups are shown in Scheme 17, Scheme 18 and Scheme 19.

The particles bearing ATRP initiator groups were used for initiation of polymerizations of glycidyl methacrylate (GMA), PNIPAM and later were grafted with clickable 3-(prop-2-ynyloxycarbonylamino)-phenylboronic acid (PCAPBA). The process yielded boronic acid functionalized polymer brushes on the surface of silica particles [214]. PCAPBA was synthesized according to the procedure described in the literature [215]. The particles bearing the RAFT transfer agents were used in polymerization of poly(glycidyl methacrylate), poly(oligo(ethylene glycol) methyl ether methacrylate) (POEGMA), and poly(hydroxyethyl methacrylate) [215]. The polymers formed hydrophilic coronas on the particles. The finally modified particles were used for delivery of nitric oxide [215].

The gold nanoparticles coated with a thin silica layer and modified by attachment of the copolymer made of *N,N*-dimethylacrylamide (DMA), 3-(methacryloyl-oxy) propyl trimethoxysilyl (MAPS), and prop-2-ynyl prop-2-enoate (PMA) were used for covalent binding of azido-modified γ-globulins, enabling antibody-mediated targeted nanoparticle delivery [214]. Reaction of alkyne functions in the interfacial layer of nanoparticles and azide moieties on modified γ-globulins was responsible for antibody immobilization.

**Scheme 20:**
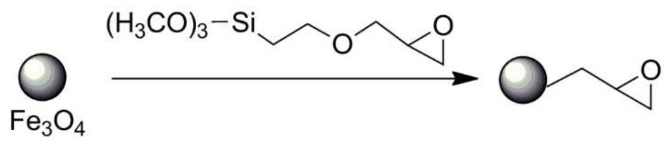
Functionalization of magnetic Fe_3_O_4_ nanoparticles introducing epoxide groups.

Trialkoxysilanes were also used for preparation of magnetic Fe_3_O_4_ nanoparticles bearing epoxy groups (see Scheme 20) [217]. However, it should be noted that the authors did not explain whether the layer containing epoxide groups was adsorbed or covalently bonded to the nanoparticles.

The nanoparticles with epoxy groups served as support for binding dendritic *p*-sulphonatocalix[(4)]arene, which could be loaded with two chemotherapeutics (doxorubicin and methotrexate) used for treatment of breast cancer [210]. The p-sulphonatocalix[(4)]arene with amine groups in the dendrimer part was covalently bound via epoxide groups of nanoparticles.

**Scheme 21:**
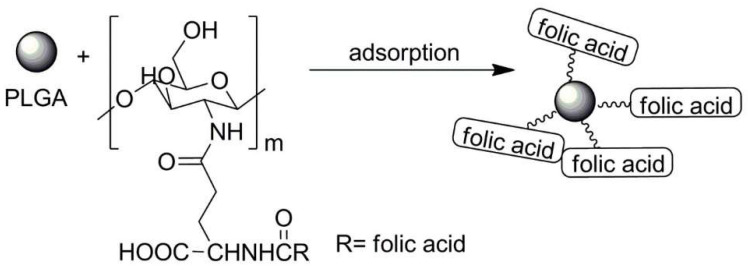
Functionalization of negatively charged PLGA nanoparticles by adsorption of chitosan bearing folic acid moieties.

Particles were also functionalized by adsorption of biomacromolecules bearing various functional groups. In some instances the adsorption was facilitated by electrostatic interactions. The negatively charged poly(d,l-lactic-*co*-glycolic acid) (PLGA) particles were readily modified by adsorption of chitosan with conjugated folic acid (see Scheme 21). The negative charge of PLGA particles was due to d-α-tocopheryl poly(ethylene glycol) succinate used during preparation of the particles by nanoprecipitation. The nanoparticles were prepared for delivery of platinum-based antitumoral carboplatin [218].

**Scheme 22:**
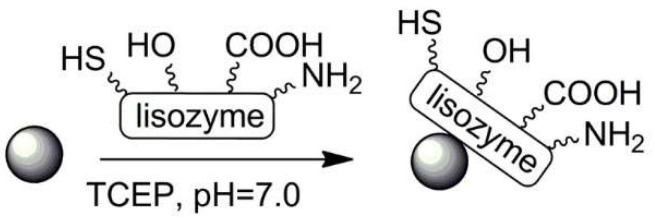
Functionalization of nanoparticles by lisozyme adsorption (TCEP denotes tris(2-carboxyethyl)phosphine buffer).

**Scheme 23:**
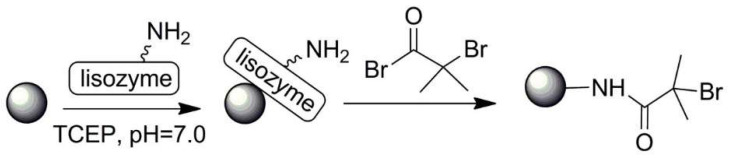
Synthesis of nanoparticles bearing bromoisobutyryl bromide moiety (ATRP initiator).

Coating nanoparticles with proteins opens possibilities for their further functionalization. For example, controlled adsorption of lisozyme onto the surface of the bare polystyrene and silica particles produced nanocarriers with carboxyl, hydroxyl, amine, and thiol groups (see Scheme 22). The two-dimensional lisozyme film coating the particles was stable at pH from 4–10 [219]. Further studies involving modification of amine groups of lisozyme film at the particles’ surface with 2-bromoisobutyryl bromide (an ATRP initiator, see Scheme 23) followed by polymerization of methacryloxyethyltrimethyl ammonium chloride provided a method of synthesis of core-shell particles with antibacterial properties [217].

**Scheme 24:**
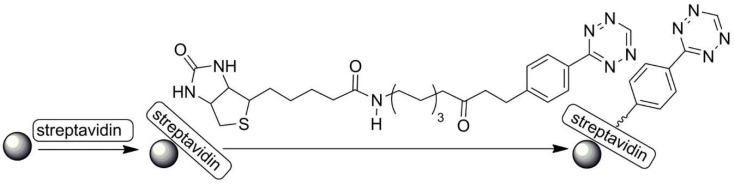
Preparation of functionalized particles by adsorption of streptavidin and complex formation between streptavidin and biotin.

Adsorption of streptavidin on the surface of microbubbles and their modification with a linker with biotin moiety at one end and tetrazine at the other end (via streptavidin–biotin complex formation) yielded microbubbles with tetrazine functions (see Scheme 24) [220]. The aforementioned method is general and could also be used for modification of polymer particles. Particles with tetrazine functional groups were used for covalent binding of antibodies bearing *trans*-cyclooctene [220]. The binding occurred via the click reaction of tetrazine and *trans*-cyclooctene groups.

Dopamine-containing compounds are very convenient as surface modification agents. Dopamine undergoes polymerization at slightly basic conditions and therefore creates adhesive films very strongly attached to polymer, metal, and ceramic surfaces.

**Scheme 25:**
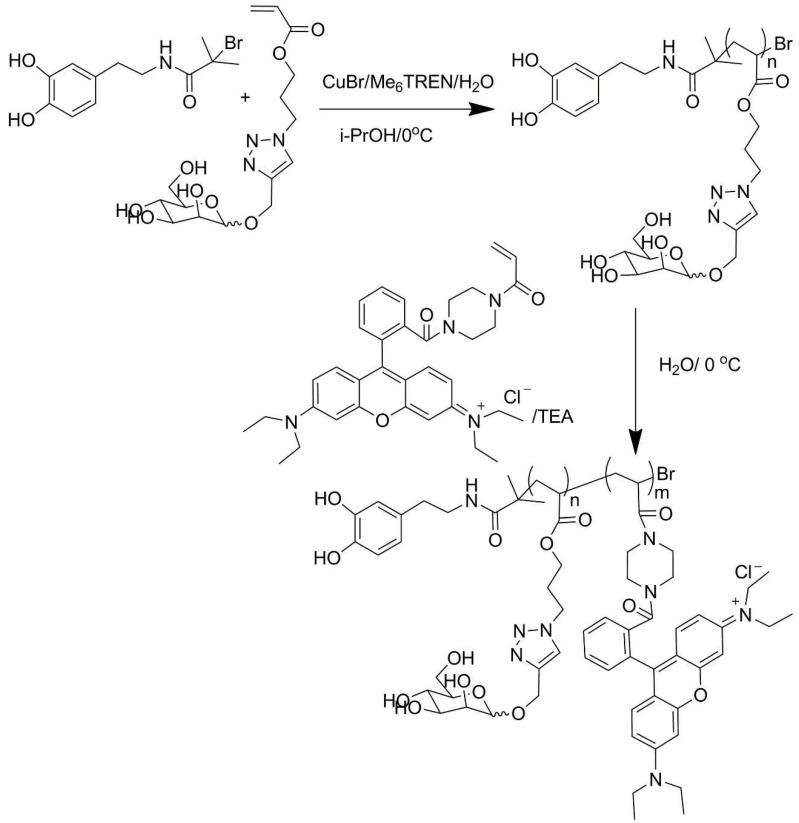
Synthesis of dopamine-poly(mannose labeled acrylate)-*b*-poly(Rhodamine B labeled acrylamide). Reproduced with permission from Reference [221].

**Scheme 26:**
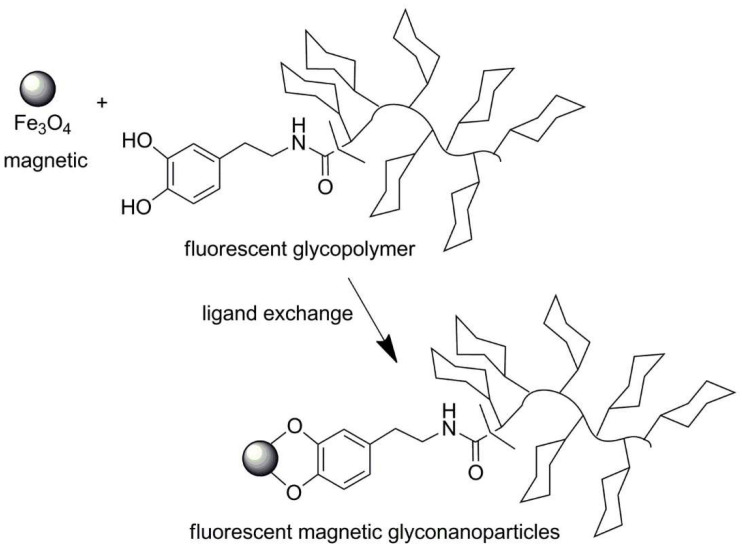
Schematic illustration of magnetic Fe_3_O_4_ nanoparticles with dopamine-poly(mannose labeled acrylate)-*b*-poly(rhodamin B labeled acrylamide). Reproduced with permission from Reference [221].

Thus, polymers bearing various functional groups labeling heir chains and dopamine end-groups could be used for easy preparation of functionalized nanoparticles. For example, fluorescent magnetic nanoparticles were obtained by coating them with copolymer labeled with Rhodamin B and equipped with dopamine end groups [221]. The copolymer was synthesized by controlled radical atom transfer block copolymerization of 2-hydroxyethyl acrylate glycomonomer (monomer with mannose labels) and rhodamine B piperazine acrylamide, using unprotected dopamine-functionalized initiator. Synthesis of copolymer and coating of particles are shown in Scheme 25 and Scheme 26. The aforementioned fluorescent nanoparticles were used for visualization of their loci in cells.

**Scheme 27:**
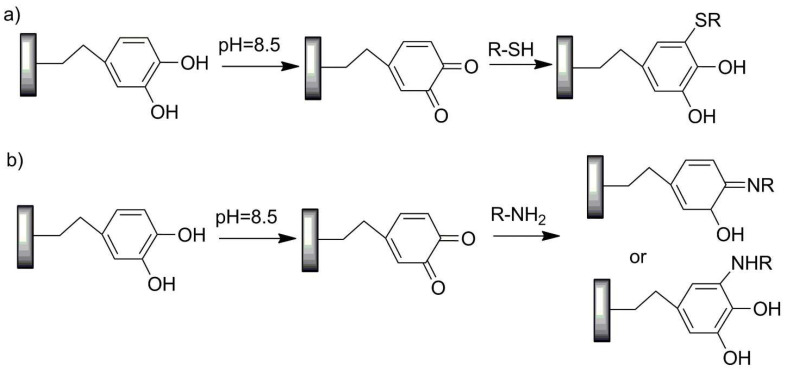
Reactions responsible for tethering of compounds with (**a**) thiol or (**b**) amine groups to polydopamine film.

Coating gold nanorods with polydopamine was used as an essential step in fabrication of nanoparticles functionalized with folic acid and Rhodamine 123 [222]. The process consisted of the following steps. First, cetyltrimethylammonium bromide (CTAB), present at the surface of the pristine nanorods as colloid stabilizer, was replaced with poly(ethylene glycol) with thiol end-groups (PEG-SH) to protect gold nanoparticles from aggregation during later steps of synthesis. Then, dopamine was polymerized in alkaline conditions (at pH = 8.5), forming shells surrounding the nanorods’ cores. The thickness of the shells was controlled by concentration of dopamine added to the reaction mixture. Subsequently, folic acid and Rhodamine 123 were immobilized on polydopamine-coated particles via amine groups of each of these compounds. It is worth knowing that the nucleophilic functional groups of ligands such as thiol or amine undergo Michael addition or Schiff base formation in these conditions (see Scheme 27) [223,224]. Presence of folic acid moieties in particle coronas enhanced their uptake by cancer cells. Labeling with Rhodamine 123 enabled visualization of nanoparticles in cancer cells. Presence of gold was essential for near infrared (NIR) mediated photothermal therapy.

**Scheme 28:**
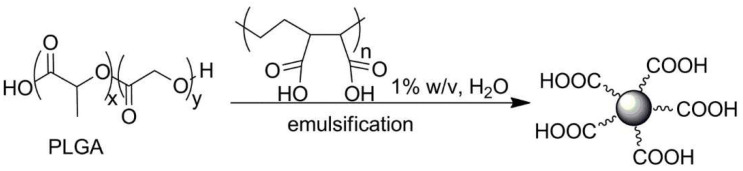
Schematic illustration of PLGA particles with carboxyl groups.

**Scheme 29:**
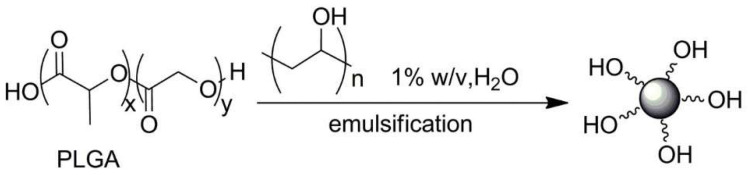
Schematic illustration of PLGA particles with hydroxyl groups.

Earlier in this subsection, we described processes of functionalization of already prepared particles. However, there is also a possibility of producing functionalized particles in a process of simultaneous particle preparation and functionalization. For example, poly(lactic-*co*-glycolic acid) (PLGA) nanospheres and microspheres with high content of hydroxyl or carboxyl groups in their interfacial layer were prepared by the oil-in-water emulsion solvent-evaporation method using methylene chloride as the oil phase and poly(vinyl alcohol) (PVA) or poly(ethylene- alt-maleic acid) (PEMA) as polymeric surface active stabilizers of PLGA/CH_2_Cl_2_ emulsion in water [225]. Preparation of functionalized PLGA particles is shown in Scheme 28 and Scheme 29.

**Scheme 30:**
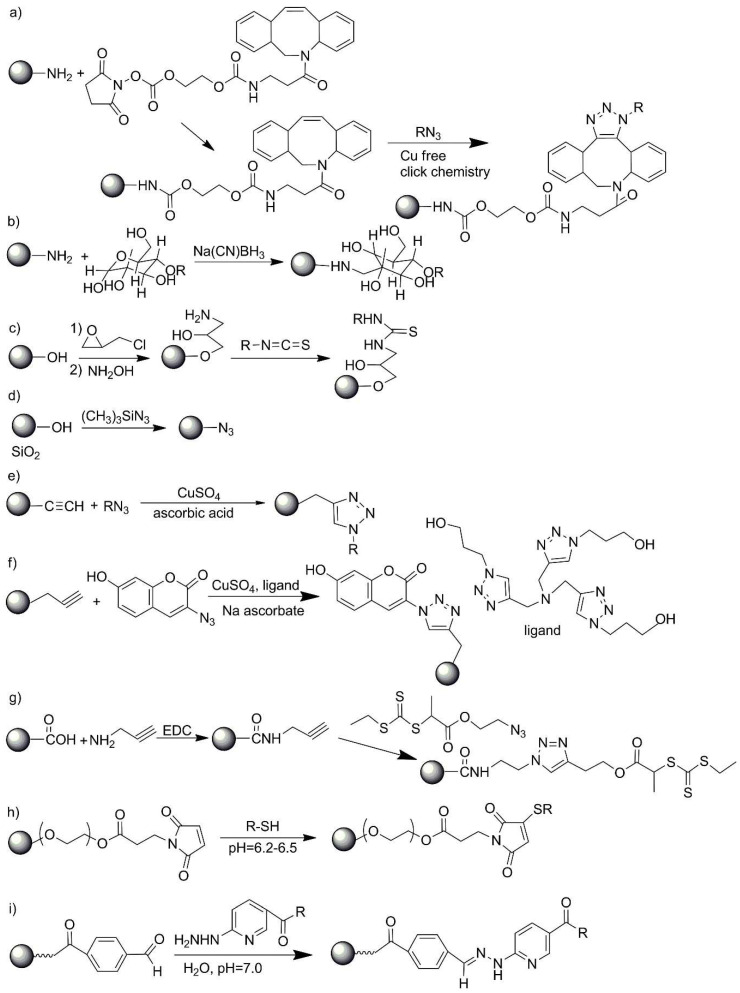
Reactions used for replacement of functional groups on surfaces of particles. Reaction conditions are provided in cited references: (**a**)—[226,227]; (**b**)—[228]; (**c**)—[229]; (**d**)—[230]; (**e**)—[216]; (**f**)—[231,232]; (**g**)—[233]; (**h**)—[234,235]; (**i**)—[212].

In many cases, the available synthetic procedures yield particles with functional groups other than those needed for particles with planned applications. The problems were usually solved by using linkers bearing at one end groups reacting with those at the particles’ surfaces and at the other end the required ones. Scheme 30 provides several examples of the “replacement” of functional groups. Details of reactions are described in cited references.

### 3.4. Nano- and Microparticles with Immobilized Ligands Specific for Nanoparticle-Selected Cell Interactions

Targeted delivery of drugs is often based on nanocarriers with surfaces equipped with ligands specifically binding to receptors on targeted cells. Many cancer cells have folic acid receptors, thus relevant carriers are equipped with folic acid residues. Some cancer cells or cells infected with bacteria or viruses display specific receptors on their cell membranes, which could be recognized by immune systems. Appropriate specific antibodies (or their Fab fragments) are used for targeting those cells.

Targeted delivery of nanoparticles is also essential in visualization of particular cells and tissues.

**Scheme 31:**
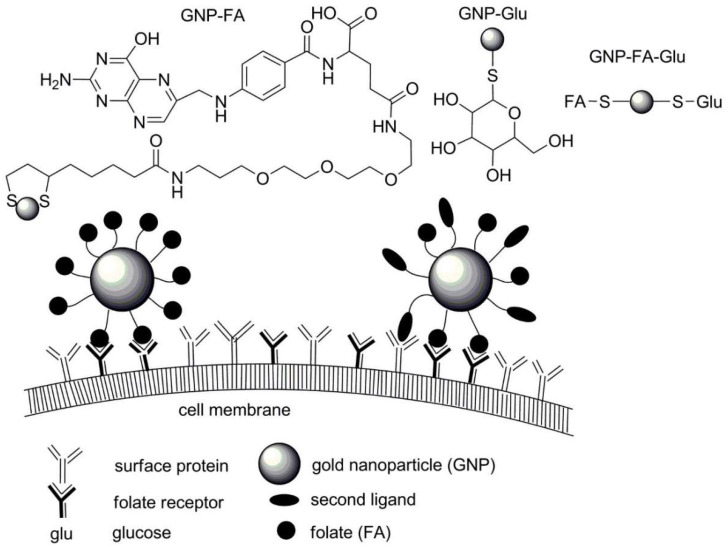
Mono- and dual ligand gold nanoparticles targeted to cancer cells. The nanoparticles were equipped with folic acid (FA) and/or glucose (glu) ligands specifically binding corresponding receptors on the cancer cells. Upper part of the scheme presents chemical structures of FA and glu (second ligand) tethered on the gold nanoparticles with sulfide linkages. Scheme is based on data from Reference [236].

**Scheme 32:**
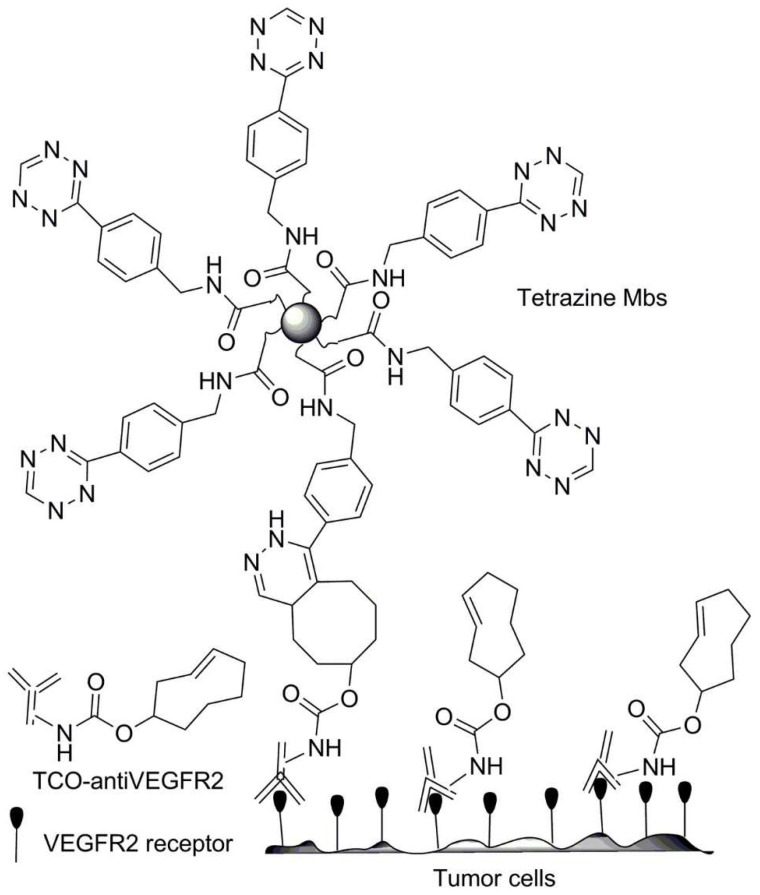
Coupling of tetrazine-tagged microbubbles (Tetrazine MBs) and intravascular VEGFR2 (endothelial growth factor) receptors on tumor cells pretargeted with specific antibodies to VEGFR2, modified *trans*-cyclooctene (TCO). Process was developed for ultrasound molecular imaging of tumor. Membrane of microbubbles composed of streptavidin (6000 macromolecules/µm^2^). Scheme prepared on the basis of data in Reference [220].

**Scheme 33:**
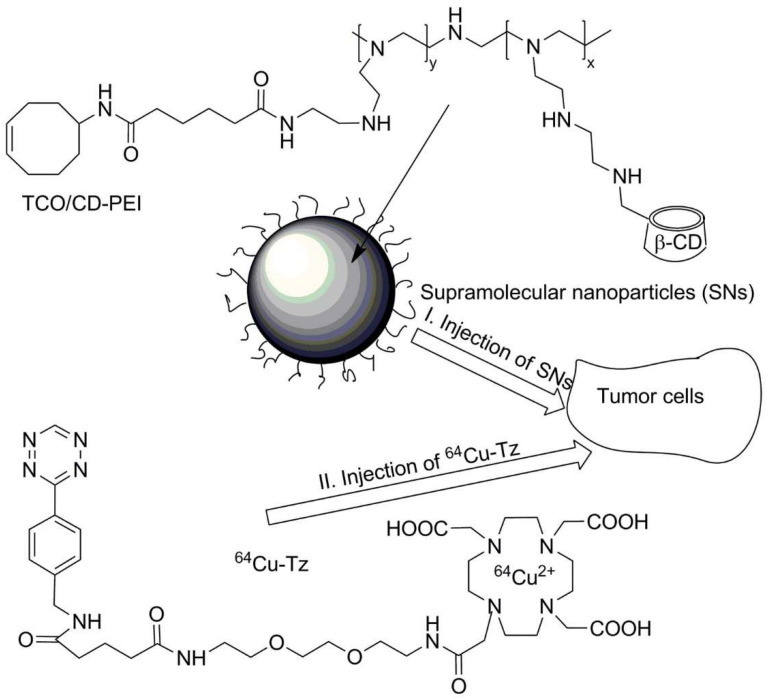
Tumor-targeting supramolecular nanoparticles (NPs) for PET (positron emission) imaging composed of trans-cyclooctene derivative of poly(ethylene imine) and *β*-cyclodextrin TCO/CD-PEI. The targeting of solid tumor eith radiolabeled ^64^Cu occurs in two steps: (I) intravenous injection of SNs, followed by (II) injection of tetrazine derivative carrying ^64^Cu (^64^Cu-Tz). In consequence of the bioorthogonal reaction between trans-cyclooctene (from TCO/CD-PEI) and tetrazine (from ^64^Cu-Tz) the conjugation of these two components takes place in vivo. The advantage of using the two-step procedure is the limit of location of radiolabeled adduct of reaction exclusive to tumor cells. The excess of unbound ^64^Cu-Tz is removed with blood circulation. Figure prepared on the basis of data in Reference [237].

**Scheme 34:**
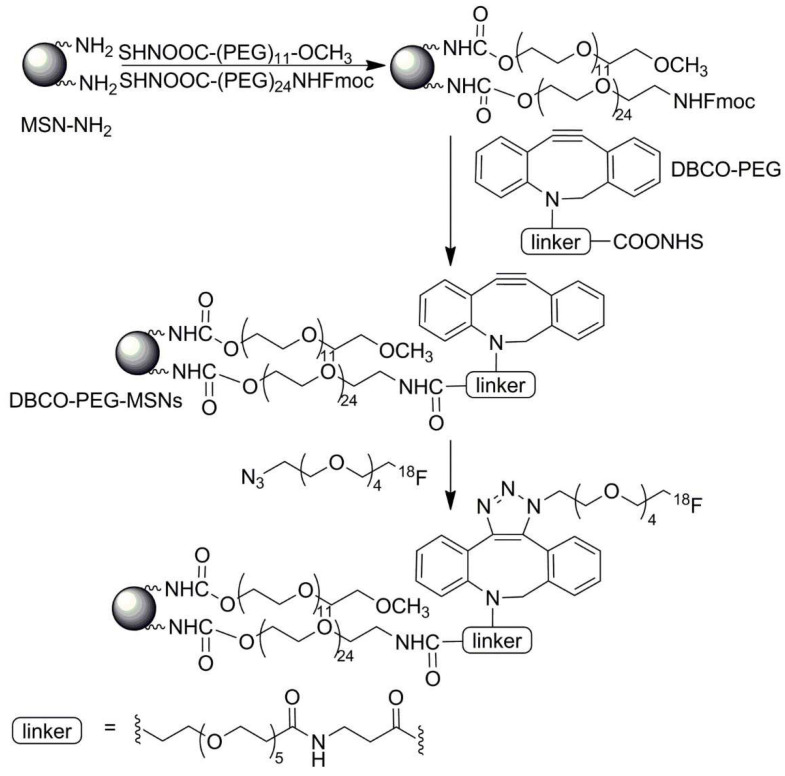
Reactions leading to functionalization of mesoporous silica nanoparticles (MSN-NH_2_) with PEG linker, followed by aza-dibenzocyclooctyne (DBCO) coupling. The reaction between DBCO-PEG-MSNs and ^18^F-labelled azide occurs under physiological-like conditions. The MSNs labeled with ^18^F were prepared for PET imaging. Scheme prepared on the basis of Reference [238].

Examples of processes used for preparation and application of the aforementioned carriers are shown in Scheme 31, Scheme 32, Scheme 33 and Scheme 34 and in Table 2.

A lot of interest was generated by complex colloidal constructs designed to function as nanocontainers and nanoreactors, often releasing drugs as a result of external stimuli. Intensively investigated systems were based on copolymers containing blocks of poly(ethylene oxide) and poly(aspartic acid). Initially these copolymers were used as building blocks for making polymeric micelles with drugs conjugated to poly(aspartic acid) chains [134,135,136,137]. Positive results of phase 1 clinical study were already published for micelles with conjugated epirubicin [245].

Recently developed nanocarriers reached a new level of smart interactions with tumor cells [246,247,248,249]. They were constructed as nanoreactors from the aforementioned copolymers with anticancer drug (camptothecin) conjugated via H_2_O_2_ sensitive linkers and bonded piperidine labels. During formation, the nanoreactors were loaded with glucose oxidase producing H_2_O_2_ in the presence of oxygen and glucose. In contact with normal tissue, the tight membrane of nanoreactors keeps them inert. However, in the neighborhood of tumor tissue, low pH triggers a cascade of events leading to the death of tumor cells. First, protonation of piperidine labels makes the membranes of nanoreactors permeable, enabling diffusion of glucose and oxygen into them. Then, glucose oxidase uses these substrates for production of hydrogen peroxide. Hydrogen peroxide cleaves the camptothecin and destroys the nanoreactors’ membranes. As a result of oxidative stress caused by hydrogen peroxide and cytotoxicity of camptothecin, the cancer cells are killed.

The example described above proves that it is possible to construct drug carriers that recognize target cells and in response release their bioactive cargo. A recent paper by Li and Kataoka strongly suggests the need for intensive and systematic studies of nanoparticle–cell interactions [249].

## 4. Conclusions

The content of the review justifies the conclusion that it was not and it will not be possible to develop a single type of nanocarrier for general usage in medicine. On the contrary, all nanocarriers should be adjusted to their particular applications. The main properties are size, chemical structure of materials constituting nanoparticles, and hydrophilicity. Today, there are methods for fabrication of organic (mainly polymer) and inorganic (most often silica and gold) particles with sizes from a few nanometers to a few micrometers. The smallest ones (up to ca. 4 nm) after fulfilling their function can be eliminated from the organism via renal pathways. The larger ones are either aggregates, which could disintegrate to particles of a few nanometers, or are made from polymers degradable to low molar mass compounds dissolved in water media and eliminated via kidney. Today there are known processes enabling fabrication of particles with hydroxyl, carboxyl, amine, thiol, and alkyne functions from pristine non-functionalized particles. It is also possible to substitute one type of functional group tethered on nanoparticles with other ones. There were elaborated routes for the most advanced methods of nanoparticle functionalization, with bioactive-specific ligands binding particles to receptors present on the membranes of specific targeted cells. The least expensive and most effective strategies for fabrication of nano- and microparticles with required functional groups seem to be prefabrication of particles loaded with bioactive compounds, particles with required size, and further functionalization with desired groups. The present state of knowledge of targeted nanoparticle delivery suggests that progress in this field would require further systematic studies on design and preparation of requests for synthetic ligands binding effectively to specific receptors on the membranes of targeted cells. Future studies should concentrate on preparation of nanoparticles long circulating with blood, able to recognize target cells, and responding to nanoparticle – target cell interaction by triggering their uptake and/or induced drug release.

## Figures and Tables

**Table 1:** Examples of synthetic and natural polymers/copolymers used for preparation of particles with/without functional groups for drug delivery systems.

No.	Chemical Composition	Functional Chemical Group(s)	Reference
Copolymers obtained exclusively via synthetic routes			
1	Poly(oligoethylene glycol) methyl ether methacrylate-co-poly(propyl methacrylate)	–OH, –COOH	[167]
2	Poly(diisopropylaminoethyl methacrylate)- poly(ethylene glycol)-poly(methacrylphosphoryl choline)	–OH, –PO_4_^2−^, –N(CH_3_)_3_	[168]
3	Poly(d,l-lactides) and copolymers with PEO or poly(2-methyl-2-oxazoline)	–OH, –COOH (after hydrolysis)	[169,170,171]
4	Poly(ethylene glycol) methyl ether-Dlabile-poly(β-amino ester)-Dlabile-poly(ethylene glycol) methyl ether	without reactive functions	[172]
5	Poly(anhydride-co-imides): poly(trimellitic anhydride-glycine/sebacic acid); poly(sebacic anhydride); poly(sebacic anhydride) and poly(1,6-bis-p-carboxyphenoxy)hexane	without reactive functions	[39,40,173]
6	Polyglycerol-co-polycaprolactone	–OH	[174]
7	Poly(tetraethylene glycolyl poly(trimethylene carbonate) grafted poly(2-nitrobenzyl methacrylate) linked by disulfide bond)-co-(5-methyl-5-propargyloxycarbonyl-1,3-dioxan-2-one); poly(ethylene glycol)-b-poly(5-methyl-5-propargyl-1,3-dioxan-2-one)	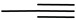	[73,175]
8	Poly(methyl-benzyloxycarbonyl) carbonate; Poly(ethylene glycol)-b-polycarbonate with benzyloxycarbonyl group; poly(ethylene glycol)-poly(2-methyl-2-benzyloxycarbonyl-propylene carbonate)	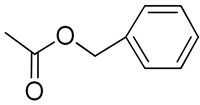	[52,70,80,81]
9	Poly(ethylene glycol)-poly(2-methyl-2-benzyloxy-methylene carbonate);	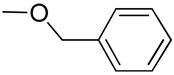	[79]
10	Poly(ethylene glycol)-poly(2-methyl-2-carbonyl-oxy-methylene alkyne carbonate);	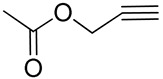	[73,78]
11	Poly(trimetylene carbonate) with 4,5-dimethoxy-2-nitrobenzyl group	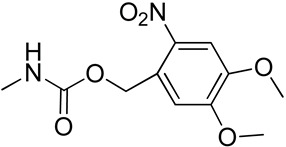	[76]
12	Poly(ethylene glycol)-b-polycarbonate with catechol bearing moiety	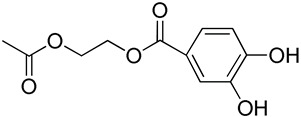	[75]
13	Poly(ethylene glycol)-b-polycarbonate with benzyloxy-p-chloromethyl group in each repeating unit	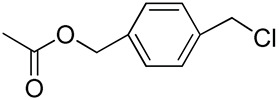	[74]
14	Poly(3,4-dihydroxybutyric acid carbonate)	–COOH	[176]
15	Poly(ethylene glycol)-b-poly(5-allyloxycarbonyl-trimethylene carbonate)	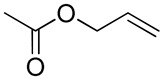	[72,177]
16	Poly(ethylene glycol)-b-poly(4-(hydroxymethyl) phenylboronic acid pinacol ester carbonate)	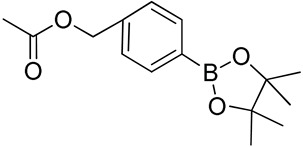	[178]
17	Poly(trimethylene carbonate) triol functionalized vinyl sulfone	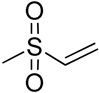	[71]
18	Poly(ethylene glycol)-b-poly(trimetylene-3-hydroxypropoxybenzaldehyde)	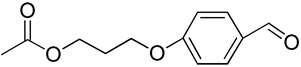	[179]
19	Poly(ethylene glycol)-b-polycarbonate functionalized urea	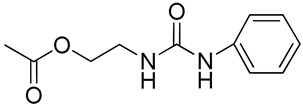	[66]
20	Poly(ethylene glycol)-b-2-(2,4- dinitrophenylthio)ethyl-2-oxo-1,3-dioxane-5-carbonate	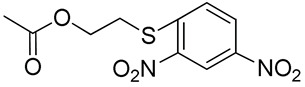	[64]
21	Poly(ethylene glycol)-b-cholesteryl 2-(2-oxo-5-carboxyloyloxy)ethyl polycarbonate	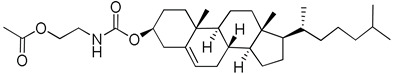	[63]
22	Polycarbonate ester-co-poly(ε-caprolactone-co-9-phenyl-2,4,8,10- tetraoxaspiro-[5,5]undecane-3-one) containing hydroxyl groups	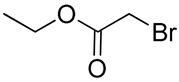 –OH	[61]
23	Polycarbonate bearing carbohydrate function	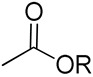 R-diacetonide sugar	[60]
24	Poly-α,β-(*N*-(2-hydroxyethyl)-l-aspartamide)-g-poly(1,3-trimethylene carbonate)	–(CH_2_)_2_OH	[180]
Natural polymers and copolymers (and/or natural polymers conjugated with synthetic polymers)			
25	Proteins and proteins linked with oligosaccharides	–COOH, –NH_2_, –OH	[181,182,183,184,185,186,187,188,189,190,191,192]
26	Functionalized chitosan-substitution of amine group of chitosan’ monomer unit in oligosaccharide chain in position R_1_: leucine conjugated chitosan; (5β-cholanic acid) glycol chitosan; octanoyl functionalized chitosan; thioglycolic acid conjugated chitosan; urocanic acid functionalized amine group of chitosan; position R_1_—salbutamol group; position R_2_—guanidine group	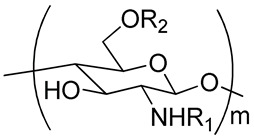 (a) 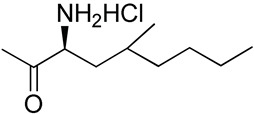 R_2_ = H (b) 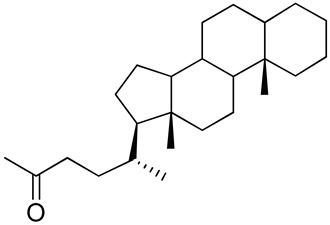 R_2_ = CH_2_CH_2_OH (c) 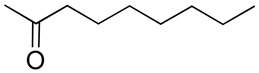 R_2_ = H or CO(CH_2_)_6_CH_3_ (d) 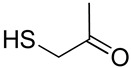 R_2_ = CH_2_CH_2_OH (e) 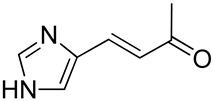 R_2_ = H (f) 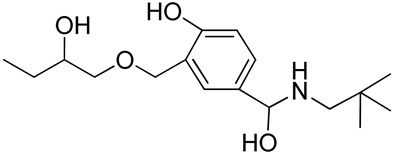 R_2_ = 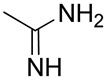	[193,194,195,196,197,198,199]
27	Apolipoproteins e.g., 1,2-dimyristoyl-sn-glycero-3-phosphocholine	–NH_2_, –OH, –PO_4_^2−^, –N(CH_3_)_3_	[200]
28	Nucleic acids with synthetic polymers	–OH, –NH_2_, –PO_4_^2−^	[201]
29	Oligosaccharides: dextran, cyclodextrins	–OH	[202,203,204,205]
30	Synthetic polymers copolymerized with oligosaccharides: Simple sugars conjugated with PEO-PPO; Pullulan-*b*-poly(*N*-vinylpyrrolidone); Alginate-*g*-poly(oligoethylene glycol methacrylate); PDMAEMA-βCDs; Poly(ethylene glycol)-*b*poly(glycidyl methacrylate) with βCD tags; Folic acid-poly(6-O-methacryloyl-d-galactopyranose)-b-poly(2-diisopropylamino)ethyl methacrylate-co-pyridyl disulfide methylacrylate;	(a) –OH (b) –OH (c) –OH, –COO- (d) –OH, –N(CH_3_)_3_^+^Cl^−^ (e) –ethylene oxide, –OH (f) –OH, –NH_2_, –COOH	[206,207,208,209,210,211]
31	Dihydrolipoic acid-poly(ethylene glycol) shell QDs ended 4-formyl benzoyl group	–CHO	[212]

**Table 2:** Examples of particles carrying attached ligand(s) binding to receptors of targeted cells.

Type (Material) of Particle	Attached Ligand	Target Cells, Tissue, Tumor, Factor in the Body, Disease, etc.	Reference
PEGylated silica mesoporous nanoparticles with Dibenzocyclooctyne (DBCO)	[(18F)]fluoro pentaethylene glycolic azide	Solid tumor	[238]
Supramolecular nanoparticles composed of poly(ethylene imine)	Trans-cyclooctene (TCO)	Solid tumor	[237]
Liposomes	Muromonab-CD_3_ (monoclonal antibody)	Autoimmune disorder	[239]
	Fab fragment of antibody		[240,241]
Poly(glycidol methacrylate) particles loaded with Docetaxel	Transferrin	Membrane bound transferrin receptors on prostate cancer	[242]
Liposomes	Internalizing RGD (arginine-glycine-aspartate) motif	α_√_β_3_ integrin receptor on angiogenic endothelial cells	[241]
Gold nanoparticles with carboxyl ended linker	Anti-17β-estradiol IgG antibodies	17β-estradiol	[243]
Gold nanoparticles with dual functionalities	Glucose and folic acid	Folate receptor/epidermal growth factor receptor on cancer cells	[236]
Functionalized microbubbles	Tetrazine	Endothelial growth factor intravascular VEGFR2 receptors and introduced bound antibodies (TCO-anti-VEGFR2)	[220]
Magnetic supraparticles core and poly-(methylacrylic acid-co-*N,N*-bis(acryloyl) cystamine) shell nanoparticles with streptavidin	Biotin labeled multiple targeting ligands	Folate and integrin receptors of HeLa cells	[244]

## Data Availability

The data presented in this study are available upon request from the corresponding author.
